# Long-Term results of total Hip Arthroplasty performed using a cementless expansive Acetabular Cup and Spotorno Femoral Stem

**DOI:** 10.12669/pjms.37.1.3089

**Published:** 2021

**Authors:** Engin Carkci, Ayse Esin Polat, Yusuf Ozturkmen, Tolga Tuzuner

**Affiliations:** 1 Engin Carkci, Department of Orthopedics and Traumatology, Istanbul Training and Research Hospital, Istanbul, Turkey; 2 Ayse Esin Polat, Department of Orthopaedics and Traumatology, Dr. Akcicek State Hospital, Kyrenia, Turkish Republic of Northern Cyprus; 3 Yusuf Oztürkmen, Department of Orthopedics and Traumatology, Istanbul Training and Research Hospital, Istanbul, Turkey; 4 Tolga Tuzuner, Department of Orthopaedics and Traumatology, Acibadem Bakirkoy Hospital, Istanbul, Turkey

**Keywords:** Hip arthroplasty, Osteolysis, Spotorno, Survivorship

## Abstract

**Objective:**

In this study we aimed to investigate the long-term clinical and radiological results, revision rates and causes, and the rate of implant survival in total hip arthroplasty performed using CLS^®^ expansion cup and Spotorno^®^ cementless femoral stem.

**Methods:**

Clinical results of total hip arthroplasty performed on 131 hips of 114 patients in Istanbul Training and Research Hospital between 1993 and 2003 were retrospectively evaluated according to the Harris Hip Score. Revision rates were determined and implant survival rates were identified using the Kaplan-Meier estimator.

**Results:**

Of the patients, 39 were males and 75 were females. The average age of the patients at surgery was 48.7±11.3 years. Patients were followed up for a mean period of 13.9±2.4 years. The mean Harris Hip Score was 34.35±6.09 preoperatively and 88.20±7.11 at the final follow-up (p<0.001). The Kaplan-Meier survivorship estimate for the cup at 13.9 years, taking revision for any reason as the end point was 95.6% (95% CI), while the 15^th^ and 17^th^ year survival rates were 90% and 85%, respectively.

**Conclusion:**

In total hip arthroplasty using a cementless expansive acetabular cup, a 95.6% survival rate is achieved after an average of 14 years, whereas the rate decreases to 85% after 17 years. Even if the incidence of cup breakage is reduced with proper implantation, particle disease and periacetabular osteolysis remains a problem for the long-term survival.

## INTRODUCTION

Increased revision rates in cemented acetabular systems have led to the development of cementless systems, placing them in the first place of choice. The purpose of the use of cementless systems is to reduce the rate of periprosthetic osteolysis and implant loosening, as well as to facilitate revision surgery if necessary, by preserving bone stock.^1^

In the early 1990s, the cementless Spotorno^®^ (CementLess Spotorno, CLS) system in total hip arthroplasty was one of the safely used options in the early 1990s. In the literature, although other cementless acetabular systems have been studied in detail, the number of studies on the CLS system acetabular component with an expansive cup is limited.^1^-^4^ Although the expansive acetabular component has advantages such as providing rotationally stable retention compared to other porous coated non-cemented acetabular components and not requiring fixation with screws, it is not widely preferred today due to the risk of fracture development in the component wing.^5^,^6^ In a recent study, the cementless Spotorno^®^ femoral stem, even in young patients, has been reported to have a survival rate of 91.2% after 18 years, when all causes are considered, and the rate reaches 95.1% when infection is excluded.^7^ With the femoral stem of the CLS system having such a high survival rate, we wanted to determine the survival rate of the expansive acetabular component and the total prosthesis.

In this study, we aimed to investigate the long-term clinical and radiological results, revision rates and causes, and the survival rate in total hip arthroplasty (THA) using expansive acetabular cup.

## METHODS

After getting the approval from the Ethics Committee of Istanbul Training and Research Hospital (Ref. 334 dated Sept. 20, 2013), 278 patients who had undergone THA using CLS expansion type acetabular components and Spotorno^®^ cementless femoral stems (Protek AG, Bern, Switzerland) between 1993 and 2003 were retrospectively evaluated. Patients with dysplastic coxarthrosis that required femoral shortening, patients with a history of stroke or malignancy that would affect the clinical outcomes during follow-up, and those who did not agree to or were unable to participate in the study were excluded. Finally, 131 hips of 114 patients (75 females [86 hips], 39 males [45 hips]), with a mean age of 48.7±11.3 years (range: 18 to 68 years) at the time of THA, were available for clinical and radiological assessments with a minimum follow-up of 10 years. Etiological assessment of the THAs revealed that the most common cause of surgery was primary osteoarthritis with 52.7%, followed by developmental hip dysplasia (DDH) with 23.7%, osteonecrosis of the femoral head with 9.2%, posttraumatic arthritis with 5.3%, rheumatoid arthritis with 5.3%, and arthritis is secondary to ankylosing spondylitis with 3.8% of the hips.

**Fig.1:**
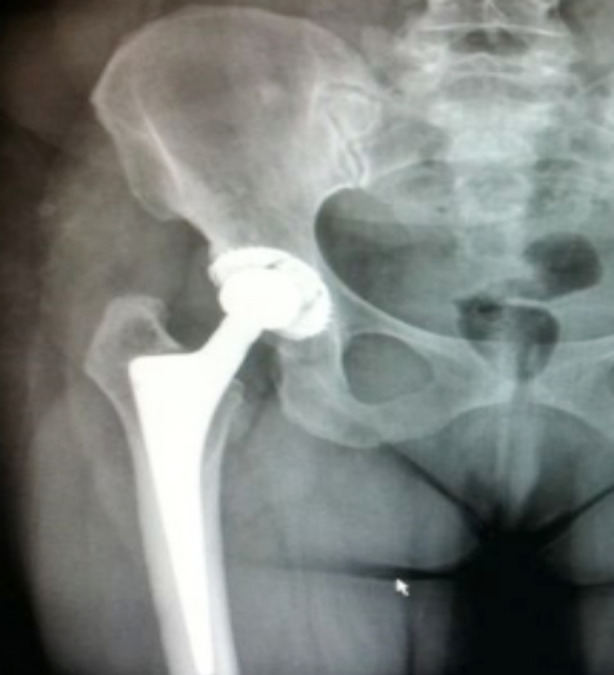
Radiograph of a 64-year-old female patient, showing the 15-year-old implant in THA. The patient had a Harris Hip Score of 96.

All surgeries were performed with a posterolateral incision in the lateral decubitus position. The acetabular component was implanted using the expansion technique described by Spotorno et al.^5^ The femoral component in the CLS system is a prosthesis with a metaphyseal fixation and is made of high-strength titanium (Ti-6AI-7Nb) with wedge-shaped tapers at a collodiaphyseal angle of 145° on the three planes. The prosthesis provides rotational stability thanks to its triangular design. The acetabular component, on the other hand, contains six star-shaped lobes made of titanium (Ti-6AI-7Nb) that have a hemispherical shape and shrink as they near the center. The fixing teeth are placed radially in three different latitudes. The spinous protrusions and the expansion feature of the acetabular component have a rotational stability-enhancing feature and increases osseointegration thanks to its tight surface contact. Acetabular expansive cups ranging from size 44 to 58 and femoral stems with sizes ranging from 7 to 15 were used on our patients (Fig.1).

Patients’ demographics and postoperative complications were provided from medical records. Clinical evaluations according to the Harris Hip Score (HHS) were made by questioning the patients before surgery and at the last follow-up. For preoperative planning, anteroposterior pelvic X-ray and anteroposterior and lateral radiographs of the hip and the femur were taken for all patients. At the final follow-up, radiological evaluations were made by taking anteroposterior radiographs of the pelvis and lateral radiographs of the hip.

In evaluating the acetabular component, the acetabulum was divided into three zones as described by DeLee and Charnley.^8^ The presence and width of any radiolucent zone in these zones were calculated. In addition, three measurements were made to assess the acetabular component stability. First, the angle between the line connecting both ends of the acetabular component on the joint side and the line connecting each tear drops in the anteroposterior pelvic radiographs was measured to determine the acetabular cup angle. Second, the distance between the lower corner of the acetabular component and the line connecting both tear drops was measured to evaluate vertical migration. Third, the distance between the center of the outer wall of the acetabular component and the Köhler line (ilioischial line) was used to evaluate horizontal migration.^9^ The presence of any of the following conditions was considered as an evidence of acetabular loosening; continuous radiolucencies around the cup in Zones one to three according to DeLee and Charnley,^8^ a vertical or horizontal migration of >5 mm, a tilt of >5° of the acetabular component compared to the initial postoperative radiograph, or a breakage of the cup. Evaluation of heterotopic ossification was made according to the Brooker classification.^10^

### Statistical analyses

The SPSS v.15.0 (SPSS Inc., Chicago, IL, USA) software was used in statistical analyses. Descriptive statistics were presented as number and percentage for categorical variables, and as mean, standard deviation, minimum and maximum for numerical variables. Numerical variables were evaluated using the Mann-Whitney U test since the differences between the groups did not exhibit a normal distribution. Intergroup comparisons of the categorical variables were made with the chi-square analysis. In cases if conditions were not met, Monte Carlo simulation was utilized. Survival rates were determined by the Kaplan-Meier analysis.

## RESULTS

Of the patients, 39 (34.2%) were males and 75 (65.8%) were females. Forty-seven patients were operated on their right hip, 50 were operated on the left hip and 17 (14.9%) patients on both hips. Surgery of the patients with bilateral hip problems was performed in different sessions at least three months apart, prioritizing the hip that had more complaints. The average age of the patients at the time of surgery was 48.7±11.3 years (range: 18 to 68 years). Patients were followed up for a mean period of 13.9±2.4 years (range: 10 to 20 years) (Table-I).

**Table-I:** Demographic information of the patients.

Gender, n (% patient)	Male	39 (34.2)
	Female	75 (65.8)
Age, Mean±SD (min-max)		63.2±11.4 (29-79)
Age range, n (% patient)	<30	1 (0.9)
	30-39	0 (0.0)
	40-49	12 (10.5)
	50-59	30 (26.3)
	60-69	26 (22.8)
	>=70	45 (39.5)
Age at surgery, Mean±SD (min-max)		48.7±11.3 (18-68)
Age range at surgery, n (% hip)	<20	1(0.8)
	20-29	9 (6.9)
	30-39	20 (15.3)
	40-49	35 (26.7)
	50-59	38 (29.0)
	>=60	28 (21.4)
Etiology, n (% hip)	Primary osteoarthritis	69 (52.7)
	DHD	31 (23.7)
	AVN	12 (9.2)
	FNF pseudoarthrosis	7 (5.3)
	Rheumatoid arthritis	7 (5.3)
	Ankylosing spondylitis	5 (3.8)

**AVN:** avascular necrosis, **FNF:** femoral neck fracture, **DHD:** developmental hip dysplasia, **SD:** standard deviation.

The mean HHS was 34.35±6.09 preoperatively and 88.20±7.11 (range: 69 to 100) at the final follow-up (p<0.001). At the final follow-up, 63 (48.1%) of the hips had excellent scores (100-90), 44 (33.6%) had good (89-80), 20 (15.3%) had fair (79-70) and four (3.1%) had poor (<70) scores.

**Fig.2:**
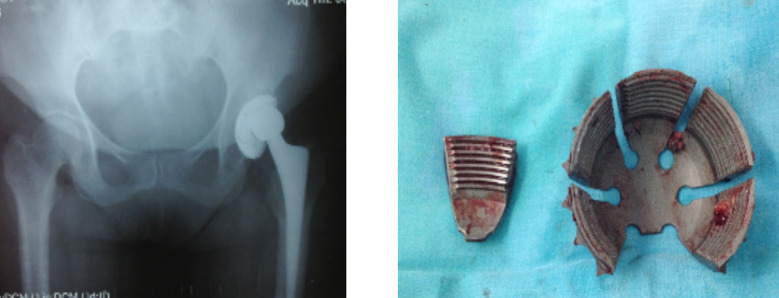
(a) Implant radiograph and photo of a 53-year-old female patient. The patient underwent revision surgery with the diagnosis of acetabular cup breakage in the 12^th^ year of THA. (b) The broken wing of the acetabular cup during revision surgery.

Revision surgery was performed on 12 hips (9.2%) due to broken shell of the cup in four cases (Fig.2), aseptic loosening in three, polyethylene wear in three, late periprosthetic infection in one, and late dislocation that included polyethylene wear and aseptic loosening in one hip. Femoral stem revision was performed in three hips (2.3%), of which two had aseptic loosening and one had an infection. Factors affecting the revision surgery and a brief comparison of the patients who underwent revision surgery versus those who did not are presented in Table-II.

**Table-II:** Some of the factors that affected revision surgery.

		*Revision*		

		None	Yes	p
Gender, n (%)	Male	41 (34.5)	4 (33.3)	1.000
	Female	78 (65.5)	8 (66.7)	
Age at the time of surgery, Mean±SD		48.9±11.3	46.8±12.1	0.505
Age range at the time of surgery, n (%)	<20	1 (0.8)	0 (0.0)	0.285
	20-29	7 (5.9)	2 (16.7)	
	30-39	19 (16.0)	1 (8.3)	
	40-49	33 (27.7)	2 (16.7)	
	50-59	32 (26.9)	6 (50.0)	
	>=60	27 (22.7)	1 (8.3)	
Harris Hip Score, Mean±SD		89.6±8.4	73.7±3.4	<0.001
Duration of follow-up, Mean±SD		13.8±2.4	15.3±2.4	0.063

**SD:** standard deviation, Significant p-values are written in bold.

When the radiolucent areas around the acetabular component in the remaining 119 hips were examined, a radiolucent zone not exceeding 2 mm was detected in Zone-I in eight hips, in Zone-II in four hips and in Zone 3 in five hips, according to DeLee and Charnley’s classification. The mean acetabular cup angle was 46.5° (range: 30° to 65°) at the final follow-up. At the final follow-up, none of the patients who have not undergone a revision surgery exhibited a tilt or horizontal and vertical migration of more than 2 mm of the acetabular cup, which would suggest acetabular cup instability. The mean acetabular cup angle of the patients who underwent revision surgery was 53.0°±9.5° at the final follow-up. In four patients with broken cup wings, the mean acetabular cup angle was 55.75°±6.45° at the final follow-up.

Based on the Brooker classification, Grade-I heterotopic ossification was present in 11 hips, Grade-II in four hips, and Grade 3 in two hips. None of these patients had hip pain, while two patients with Grade 3 heterotopic ossification had limited hip movements.

Various complications that did not require a revision surgery were detected in 21 patients (18%). Dislocation developed in three patients within the first month after surgery. It was noticed that these patients were followed up with hip abduction orthosis for three weeks after closed reduction, and they did not have any stability problems afterwards. Three patients that developed deep vein thrombosis, diagnosed by clinical examination and Doppler ultrasonography, recovered with medical treatment. Femoral fractures developed during the placement of the femoral component in five patients, one of which was in the greater trochanter. Fractures were fixed with cerclage wiring. The prostheses in these patients were found to be stable at the final follow-up. Superficial wound infection in six patients and deep wound infection in another one were taken under control with antibiotics and wound debridements. One late deep periprosthetic infection underwent revision surgery. Two patients were observed to develop foot drop due to sciatic nerve damage in the postoperative period. One of these patients fully recovered at the 3^rd^ and the other at the 6^th^ month, after being followed up with ankle-foot orthoses.

**Fig.3:**
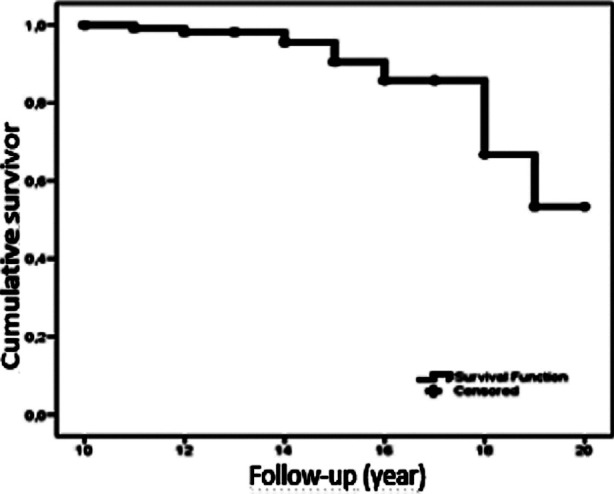
Cumulative survival rate in hip arthroplasty according to the time spent for follow-up.

The Kaplan-Meier survivorship estimate for cup, taking revision for any reason as the end point was 95.6% (95% CI) at 13.9 years, while the 15th and 17th year survival rates were 90% and 85%, respectively (Fig.3).

## DISCUSSION

The most important aspect of our study is that it contains reliable results about the complications, causes of revision, and implant survival rates in THA performed using expansive cup, thanks to its size of patient population and follow-up period that ranged from 10 to 20 years.

Spotorno^11^ stated that the 16-year survival results for the acetabular component was 91.7% and for the femoral stem was 98.6%. The authors performed 186 revision surgeries in 186 of the 6,248 hips (7.5%) that were placed a CLS system between 1983 and 1999. The causes of revision were a broken cup in eight hips, polyethylene wear in four and aseptic loosening in two. Smeekes et al. reported that in the long-term follow-up of 120 patients who underwent CLS system, the implant survival rate due to any reason was 72.8% and the survival rate due to aseptic acetabular cup loosening was 80.1%.^4^ De Witte et al.^1^ performed a total of 14 revision surgeries (due to cup in 10, femoral stem in three, infection in one) in their series of 102 cases with an average follow-up period of 142 months and reported the 10-year implant survival rate as 92.2% and the 15-year rate as 78.4%. The authors found that acetabular cup loosening was due to high polyethylene wear rates and that factors such as using a femoral head of 32 mm, male gender, a high body mass index, and young age at first surgery increased polyethylene wear.

Rozkydal et al.^12^ reported a 92% clinical survival rate for the acetabular expansive cup at the end of 15 years, as in 112 hips of the 105 patients they evaluated, they performed revision surgeries in seven hips (6.3%); due to aseptic loosening of the acetabular components in two hips, cup breakage in three, and polyethylene wear in two. The authors asserted that the expansive cups showed good results even after 15 years and the reason for revision in the CLS system was generally due to the acetabular component. The authors also found that the cup breakage was caused by failure of the acetabulum in the proximal and lateral regions, especially in dysplastic hips. In a recent study, in the hybrid total hip prosthesis performed using uncemented CLS cup and cemented femoral stem, 86.1% survival was achieved in 20.3 years of follow-up.^13^ In our study, in accordance with the literature, 95.6% survival was achieved in the mean follow-up period of 13.9 years.

In components where primary fixation is properly made, osseointegration on porous surfaces reaches its highest level after 8 to 12 weeks. Therefore, the patients were not allowed full weight-bearing for 8 to 12 weeks. The expansion feature of the expansion type acetabular component increases rotational stability and enhances the osseointegration by creating a tight contact surface with its spinous protrusions that develop after fixation.^5^ The low rates of osteolysis detected in acetabular components during long-term follow-ups support this statement. Another advantage of the CLS systems is that since no screws are used in fixation of the acetabular system, the risk of damaging nervous-vascular structures is very low.

In addition to the surface properties of the acetabular component, the acetabular component geometry also plays an important role in the formation of radiolucent zones around the acetabular component.^14^ Due to the differences in geometric structures, different osseointegration areas appear around the component. This leads to the creation of radiolucent areas of different proportions that do not cause instability around the implant. In our study, when the radiolucent zones around stable acetabular components were evaluated in 119 hips that had not undergone revision surgery, radiolucent zones not exceeding 2 mm in different zones were detected in 17 (14.3%) hips according to DeLee and Charnley classification. In this sense, our results were in line with those of the literature.

Survival of the acetabular component depends on primary stability. It is critical to ensure proper cup anteversion by paying attention to anatomical landmarks such as transverse acetabular ligament.^15^ Technical errors during implantation can cause early loosening. The expansive cup enhances the bone growth and osseointegration after primary stability, and provides an excellent attachment. Problems that may occur around this period may cause later loosening and wing breakages of the cup. Although Heilpern and Parker^6^ detected a cup breakage in six patients that were placed an expansive cup, they reported that the incidence of a cup breakage could be higher despite having failed to detect it radiologically, especially in patients with hip pain. The authors reported that the lateral placement of the acetabular component may cause a broken cup by increasing the load on the wings and therefore abandoned using this implant due to this complication. The most frequent reason for revision surgery in expansive cups that we encountered was a broken wing. We found that the mean abduction angle of the acetabular cup was higher than 55 degrees in patients who underwent revision surgery after wing breakage. We believe that wing breakage is caused by metal fatigue that develops due to excessive load on the wings. Acetabular failure in dysplastic hips is also an important risk factor for component loosening. Today, the prevalence of cup breakages, which is shown as the main reason for not using an expansive cup, is very low and the implant survival rate is comparable to other porous-coated acetabular components.^16^-^18^ Two of the three patients who underwent revision surgery due to polyethylene wear were males and they had undergone their first surgery at a young age. We believe that the more active life style of men compared to women can increase polyethylene wear and loosening. However, it should be kept in mind that preoperative planning should be properly performed, considering that the primary factor for long survival of a prosthesis is primary stability during implantation.^19^

### Limitations of the study

The retrospective design and execution of the surgeries by different surgeons are the limitations of our study. However, this study is among the few ones in the literature, considering its patient population size, long follow-up period, and conduction in a single center.

## CONCLUSION

In conclusion, although rarely used today, we achieved good clinical results in THA using cementless expansive acetabular cups, with a 95.6% rate of implant survival after an average follow-up period of 14 years. The major causes of acetabular component failure are cup breakages and osteolysis. Even if the incidence of cup breakage is reduced with proper implantation, particle disease and periacetabular osteolysis remains a problem for the long-term survival.

### Authors` Contribution:

**EC and AEP** conceived, designed, did statistical analysis, data collection and manuscript writing.

**YO** and **TT** did review and final approval of manuscript.

**AEP** is responsible and accountable for the accuracy or integrity of the work.
